# Risk Factors for Active Trachoma and Ocular *Chlamydia trachomatis* Infection in Treatment-Naïve Trachoma-Hyperendemic Communities of the Bijagós Archipelago, Guinea Bissau

**DOI:** 10.1371/journal.pntd.0002900

**Published:** 2014-06-26

**Authors:** Anna R. Last, Sarah E. Burr, Helen A. Weiss, Emma M. Harding-Esch, Eunice Cassama, Meno Nabicassa, David C. Mabey, Martin J. Holland, Robin L. Bailey

**Affiliations:** 1 Clinical Research Department, Faculty of Infectious and Tropical Diseases, London School of Hygiene and Tropical Medicine, London, United Kingdom; 2 Disease Control and Elimination Theme, Medical Research Council Unit, The Gambia, Fajara, The Gambia; 3 MRC Tropical Epidemiology Group, Faculty of Epidemiology and Population Health, London School of Hygiene and Tropical Medicine, London, United Kingdom; 4 Programa Nacional de Saúde de Visão, Ministério de Saúde Publica, Bisssau, Guiné Bissau; University of California San Francisco, United States of America

## Abstract

**Background:**

Trachoma, caused by ocular infection with *Chlamydia trachomatis*, is hyperendemic on the Bijagós Archipelago of Guinea Bissau. An understanding of the risk factors associated with active trachoma and infection on these remote and isolated islands, which are atypical of trachoma-endemic environments described elsewhere, is crucial to the implementation of trachoma elimination strategies.

**Methodology/Principal Findings:**

A cross-sectional population-based trachoma prevalence survey was conducted on four islands. We conducted a questionnaire-based risk factor survey, examined participants for trachoma using the World Health Organization (WHO) simplified grading system and collected conjunctival swab samples for 1507 participants from 293 randomly selected households. DNA extracted from conjunctival swabs was tested using the Roche Amplicor CT/NG PCR assay. The prevalence of active (follicular and/or inflammatory) trachoma was 11% (167/1508) overall and 22% (136/618) in 1–9 year olds. The prevalence of *C. trachomatis* infection was 18% overall and 25% in 1–9 year olds. There were strong independent associations of active trachoma with ocular and nasal discharge, *C. trachomatis* infection, young age, male gender and type of household water source. *C. trachomatis* infection was independently associated with young age, ocular discharge, type of household water source and the presence of flies around a latrine.

**Conclusions/Significance:**

In this remote island environment, household-level risk factors relating to fly populations, hygiene behaviours and water usage are likely to be important in the transmission of ocular *C. trachomatis* infection and the prevalence of active trachoma. This may be important in the implementation of environmental measures in trachoma control.

## Author Summary

Trachoma, caused by ocular infection with *Chlamydia trachomatis*, is the leading infectious cause of blindness worldwide. The World Health Organization elimination strategy includes community mass treatment with oral antibiotics, education regarding hygiene and facial cleanliness and environmental improvements. Population-based trachoma prevalence surveys are essential to determine whether community interventions are required. Knowledge of risk factors associated with trachoma and *C. trachomatis* infection in a particular setting may help prioritise trachoma elimination activities. We conducted a trachoma prevalence survey to establish the prevalence of active (follicular and/or inflammatory) trachoma and *C. trachomatis* infection on the Bijagós Archipelago of Guinea Bissau. We also collected household risk factor data from survey participants. Active trachoma prevalence was 11% overall and 22% in children aged 1–9 years. *C. trachomatis* infection prevalence was 18% overall and 25% in children aged 1–9 years. Active trachoma and the presence of *C. trachomatis* infection were strongly correlated. Risk factors for disease and infection were similar. In this environment, measures of facial cleanliness (ocular and nasal discharge) and household-level risk factors relating to fly populations, hygiene behaviours and water usage are likely to be important in *C. trachomatis* transmission. This may have implications in the implementation of trachoma elimination activities.

## Introduction

Trachoma is caused by ocular infection with *Chlamydia trachomatis* and is the leading infectious cause of blindness worldwide. It manifests as distinct clinical syndromes beginning with an acute self-limiting keratoconjunctivitis, which following repeated episodes may progress to a more chronic inflammatory and immunofibrogenic process leading to conjunctival scarring and blinding sequelae.

Trachoma is endemic in 50 countries, with 325 million people at risk of blinding disease [1]. Trachoma is responsible for visual impairment in 1.2 million people and 3% of blindness globally [1]. The highest prevalence of active trachoma (trachomatous inflammation-follicular (TF) and/or trachomatous inflammation-intense (TI)) is in sub-Saharan Africa and the distribution of disease is heterogeneous [2].

Ocular *C. trachomatis* is probably transmitted between individuals through direct spread from eye to eye during close contact, direct or indirect spread of infected nasal or ocular secretions on fingers or cloths (fomites) and indirect passive transmission by eye seeking flies. There is no known animal reservoir of *C. trachomatis* in endemic environments, the primary reservoir being young children.

Blinding trachoma is usually found in hot, arid, dusty regions. A recent systematic review examined studies reporting higher trachoma prevalence in savannah areas and areas of lower rainfall, and found weak but consistent evidence supporting anecdotal findings that trachoma is associated with semi-arid environments [3].

This study was conducted on the Bijagós Archipelago, a remote group of islands off the coast of Guinea Bissau with a total population estimated at 24,000 [4], where trachoma is hyperendemic. The climate and environment are not typical of trachoma-endemic areas. The islands are covered with subtropical forest and altitude does not exceed 50 m. The climate is tropical, hot and humid. The islands are surrounded by mangroves and mudflats. There is significant rainfall (average 400 mm/month) from May to November [5].

Many studies have suggested that the prevalence of trachoma is associated with environmental risk factors such as poor sanitation, access to water and latrine use [6], [7]. Eye-seeking flies (*Musca sorbens*) have also been associated with trachoma as passive vectors [8] but significant disease exists in areas where fly populations are scarce and are therefore less likely to contribute to trachoma transmission [9]. *M. sorbens* preferentially breeds in human faeces and there may be association between fly populations and lack of latrine access or use [6], [8]. Social risk factors such as migration events and crowded living conditions have also been shown to be important in transmission of *C. trachomatis* and the appearance of active trachoma [10], [11].

Clustering of disease at the community, household and bedroom levels has been noted and is likely to reflect the dynamics of transmission between family members with prolonged close contact [6], [10]–[12]. Most transmission events have been shown to occur at the household level with more gradual spread within the community [13].

The World Health Organization (WHO) advocates the implementation of the SAFE strategy (Surgery for trichiasis, Antibiotics for active infection, Facial cleanliness to prevent disease transmission and Environmental improvement to increase access to water and sanitation) for trachoma elimination. The WHO recommends annual mass treatment of entire communities with oral azithromycin for three years if the prevalence of TF in 1–9 year olds within a district or community exceeds 10%. Mass antibiotic treatment aims to clear infection from communities such that transmission ceases to be a public health concern [14]. Following this, an assessment is made of A, F and E interventions and a decision is taken to continue or cease treatment [15].

Despite their inclusion in the SAFE strategy, local environmental factors are not well understood, though many are potentially modifiable risk factors for infection and disease. The relative importance of these risk factors is not clear and may differ between communities. Fewer studies have investigated risk factors for disease and infection simultaneously [16]–[19]. Understanding risk factors associated with trachoma and *C. trachomatis* infection may increase our understanding of disease and transmission dynamics allowing for optimization of community-specific interventions.

We examined household and individual-level risk factor associations with ocular *C. trachomatis* infection and active trachoma in this unique environment, where trachoma is a significant public health problem. Prior to these surveys, these communities were treatment-naïve and had not been exposed to any trachoma control interventions.

## Methods

### Ethical Approval

This study was conducted in accordance with the declaration of Helsinki. Ethical approval was obtained from the Comitê Nacional de Ética e Saúde (Guinea Bissau), the LSHTM Ethics Committee (UK) and The Gambia Government/MRC Joint Ethics Committee (The Gambia). Verbal consent was obtained from community leaders. Written informed consent was obtained from all study participants or their guardians on their behalf if participants were children. A signature or thumbprint is considered an appropriate record of consent in this setting by the above ethical bodies.

### Study Design and Population

We conducted a cross-sectional population-based trachoma prevalence survey on four islands of the Bijagós Archipelago of Guinea Bissau (Bubaque, Canhabaque, Soga and Rubane) in January 2012. Trachoma survey methodology has been described previously [20]–[22]. We randomly sampled one in five households, representing a one stage probability sample design satisfying desired criteria for population-based prevalence surveys [20], [21]. A sample size of 1500 ensured adequate power with conservative correction (using a design effect of 4) to account for anticipated household clustering. The sample size provides over 90% power to detect an odds ratio (OR) of 2 associated with a risk factor found in 20% of subjects without disease or infection, or an OR of 3 for a risk factor present in 5% of subjects without disease or infection with 95% confidence. The sample size also provides good precision for an estimated TF prevalence of >25% in 1–9 year olds on the four islands of Bubaque and Canhabaque (±4%), Soga (±6%) and Rubane (±10%), which is adequate to determine whether these communities require mass drug treatment with azithromycin in line with WHO policy.

### Household Census

A census of persons resident in randomly selected households was conducted prior to the household survey. Residency was defined as living within the household for longer than the preceding month or intending to stay resident in the household for longer than one month. This was updated to reflect the *de facto* population (those present in the household on the previous night) to limit absenteeism.

### Household Risk Factor Survey

Demographic, socio-economic and environmental information was collected at household and individual levels. Household-level risk factor data were obtained using questionnaires administered to the household head or an appropriate responsible adult and included items on the level of education of the household head, their socio-economic status, whether the household had been exposed to any health education or promotion within the community, household access to and use of latrines, access and use of water and measures of sanitation, waste and presence of flies in the environment. The questionnaire was supported through observational data collected on water use, latrine use and environmental sanitation. Household size (measured as number of members of all ages) and number of children under the age of 10 years within the household was recorded. Researchers were masked to trachoma status of household members at the time of the household survey.

### Trachoma Prevalence Survey

Following the household risk factor survey all individuals from study households were invited to attend for clinical examination and conjunctival sampling. Individuals' age, sex and ethnic group and data on facial cleanliness (the presence of ocular and/or nasal discharge and whether or not there were flies on the face) were collected at the time of examination.

### Clinical Assessment

A single trained examiner assessed each participant using the WHO simplified grading system where TF (trachomatous inflammation – follicular) and/or TI (trachomatous inflammation – intense) constitute active trachoma and TS (trachomatous scarring), TT (trachomatous trichiasis) and CO (corneal opacity) are trachomatous sequelae which may lead to blindness [23]. A trachoma grade was assigned to the upper tarsal conjunctivae of each consenting participant using adequate light and a 2.5× binocular magnifying loupe.

### Conjunctival Sampling

Two sequential samples were taken from the left upper tarsal conjunctiva of each participant with Dacron swabs (Fisher Scientific, UK) using a standardised procedure [24], [25]. The first swab was collected into transport medium for other studies. The second dry swab was collected into a microcentrifuge tube (Simport, Canada) and used in this study. Previous work using the Roche Amplicor CT/NG assay (Roche Molecular Systems, NJ USA) in a population-based study has shown that there was good agreement between first and second swabs with respect to *C. trachomatis* DNA positivity by PCR [26]. Swabs were kept on ice in the field and frozen to −80°C within 8 hours of collection.

Measures were taken to avoid cross-contamination in the field. Control swabs (pre-marked swabs drawn at random from the swab dispenser and passed 10 cm in front of the eye ensuring no contact between the swab tip and participant) were taken to ensure field and laboratory quality control.

### Community Mass Treatment

After survey completion all communities on the study islands were treated with a single height-based dose of oral azithromycin in accordance with WHO and national protocols.

### DNA Extraction

Each swab was suspended in 400 µl sterile phosphate buffered saline (PBS) after thawing at room temperature. DNA was extracted from the swab/PBS suspension using an adapted whole blood protocol on the QIAxtractor (Qiagen, Crawley, UK) automated instrument and eluted into a final volume of 50 µl DX Elution Buffer (Qiagen).

### Detection of Infection with *C. trachomatis*

*C. trachomatis* DNA was detected using the Roche Amplicor CT/NG assay (validated for use with ocular swabs [27]). Required reaction buffer conditions were obtained as described previously and used in the standard assay [28]. Positive and negative samples were assigned according to the manufacturer's instructions. In this study, *C. trachomatis* infection is defined as the presence of *C. trachomatis* DNA by Amplicor PCR.

### Statistical Analyses

Data were double entered into a customised database (MS Access 2007). Discrepancies were resolved through reference to original data forms. Data were further cleaned prior to analysis in STATA 13 (Stata Corporation, College Station, Texas USA).

Random effects logistic regression models were used to assess the variability between villages and households assuming a three tier hierarchy to the data (at village, household and individual levels). Null models were used to examine the effect of cluster variables on the outcome using the likelihood ratio test (LRT), which if significant, provided strong evidence that between-village and household variance was non-zero. The log likelihood and the LRT were used to compare models.

Univariable associations with active trachoma (TF/TI) and infection with *C. trachomatis* were examined using two-level hierarchical random effects logistic regression, accounting for between-household variation. Covariates associated with active trachoma or *C. trachomatis* infection with p<0.10 (using the Wald test) were sequentially added to the multivariable model after *a priori* adjustment for age and gender (as categorical variables). Covariates were retained in the final model if the Wald p-value≤0.05 unless otherwise specified. Further exploration of environmental predictor variables was conducted using logistic and hierarchical random effects logistic regression models as appropriate using the same criteria. As *C. trachomatis* infection is on the causal pathway between several risk factors and active trachoma, models with and without *C. trachomatis* infection were fitted. The model including *C. trachomatis* infection provides estimates of independent associations of other risk factors with active trachoma which are not mediated through *C. trachomatis* infection.

All statistical analyses were carried out using STATA 13. Statistical significance was determined at the 5% level.

## Results

### Study Population Characteristics

From an estimated total rural population of 5,613 inhabitants on the four study islands [4], 1,511 individuals from 293 randomly selected households across 39 villages were enrolled. Of these, 1,508 had an ocular assessment and conjunctival swabs were obtained from 1,507. The median age of participants was 13 years (range 1 month–88 years) and 57% were female. The majority of participants were of the Bijagós ethnic group (*Table 1*).

**Table 1:** Study population characteristics.

	N^b^ (%)
Total rural population^a^	5,613
Study population	1,511
Female	869 (57.5)
Children <10 years	618 (40.9)
Bijagos ethnic group	1395 (92.3)
Islands	4
Villages	38
Households	293
Median number of members/household (IQR)	9 (24)
Median number of children <10 years/household (IQR)	4 (12)
Access to water source all year	1475 (97.6)
Distance to water source <15 minutes' walking time	1511 (100)
Access to a latrine	608 (40.4)
Access to private latrine	284 (18.8)

a Total rural population on Bubaque, Canhabaque, Soga and Rubane [4].

b N = total number and % = proportion. IQR = Interquartile range.

### Prevalence of Active Trachoma and *C. trachomatis* Infection

The prevalence of active trachoma in 1–9 year olds was 22.0% (95% Confidence Interval (CI) 18.9–25.5%) (136/618). The prevalence of active trachoma was highest in children under the age of 5 years (27.3% (95% CI 23.1–31.9%) (113/416)). Overall, 11.1% (95% CI 9.4–12.6%) (167/1508) of the study population had active trachoma. The relationship between trachoma and infection is shown in *Table 2*. *C. trachomatis* DNA was detected in 18.0% overall (269/1507) and 25.4% of 1–9 year olds (157/618). All 15 (∼1% of total) control swabs were negative for *C. trachomatis* DNA.

**Table 2:** The relationship between trachoma and presence of infection with *C. trachomatis* (*Ct*).

Age Group (N^a^)	Clinical Status^b^	N^a^(%)	*Ct*+^c^ (%)	*Ct*−c (%)
**All ages (1508)**	No trachoma	999 (66.2)	131 (13.1)	868 (86.9)
	Active trachoma (TF and/or TI)	167 (11.1)	103 (61.7)	64 (38.3)
	TF	152 (10.1)	97 (63.8)	55 (36.2)
	TI	29 (2.0)	24 (82.8)	5 (17.2)
	TS	357 (23.7)	51 (14.3)	306 (85.7)
**0–5 years (416)**	No trachoma	298 (72.0)	39 (13.1)	259 (86.9)
	Active trachoma (TF and/or TI)	113 (27.3)	69 (61.1)	44 (38.9)
	TF	104 (25.1)	66 (63.5)	38 (36.5)
	TI	19 (4.6)	16 (84.2)	3 (15.8)
	TS	11 (2.7)	2 (18.2)	9 (81.8)
**6–10 years (250)**	No trachoma	220 (88.0)	44 (20.0)	176 (80.0)
	Active trachoma (TF and/or TI)	2 (8.0)	2 (100)	0 (0)
	TF	25 (10.0)	20 (80.0)	5 (20.0)
	TI	2 (0.8)	2 (100)	0 (0)
	TS	7 (2.8)	5 (71.4)	2 (28.6)

a N = total number and % = proportion.

b Using the WHO Simplified Grading System where TF = trachomatous inflammation-follicular, TI = trachomatous inflammation-intense, TS = trachomatous scarring [23]. Individuals may appear in more than one clinical category.

c *Ct* DNA detected (*Ct*+) or absent (*Ct*−) by Amplicor PCR.

### Multilevel Clustering of Active Trachoma and *C. trachomatis* Infection

Null models for both active trachoma and *C. trachomatis* infection adjusted for age and gender showed significant clustering at island, village and household levels. For active trachoma, the variance estimated due to between-household clustering was 1.11 (standard error (SE) 0.17, *p<0.0001*). The between-village clustering variance was 0.75 (SE 0.16, *p<0.0001*) and between-island clustering variance was 0.50 (SE 0.28, *p = 0.0100*). For *C. trachomatis* infection, the variance estimated due to between-household clustering was 1.37 (SE 0.15, *p<0.0001*), between-village clustering was 0.89 (SE 0.14, *p<0.0001*) and between-island clustering was 0.40 (SE 0.18, *p = 0.0005*). The clustering effect was strongest at household level and models adjusting for clustering at household level only were a better fit than those including adjustment for village and island clustering. Adjusting for clustering at household level significantly improved the model versus standard logistic regression analyses (*p<0.0001*). Two-level hierarchical regression models with adjustment for household level clustering are presented in this analysis.

### Factors Associated with Active Trachoma

Univariable associations with active trachoma are presented in *Table 3*. The final multivariable model showed that active trachoma was strongly independently associated with *C. trachomatis* infection (OR = 11.2 (95% CI 6.9–18.1)), ocular (OR = 2.0 (95% CI 1.0–4.0)) and nasal (OR = 2.5 (95% CI 1.5–4.3)) discharge, male gender (OR = 1.9 (95% CI 1.2–2.9)) and being aged 0–5 years (OR = 10.2 (95% CI 5.1–20.4) compared to being >15 years of age) (Model 2, *Table 4*). There was also a strong independent association between household water access and active trachoma, such that households with access only to a traditional natural spring as a water source had an increased risk of active trachoma compared to households with access to multiple water sources (OR = 1.9 (95% CI 0.9–3.9)). The model without *C. trachomatis* infection shows stronger associations, indicating that some effect of these factors is mediated through *C. trachomatis* infection (*Table 4*). Comparison of the two models suggests that some of the effect of younger age and water source is partly mediated through *C. trachomatis* infection, but these remain independently associated with trachoma beyond this effect.

**Table 3:** Multilevel univariable random effects logistic regression analysis of factors associated with active trachoma^a^.

Variable	N (%)	cOR (95% CI)^b^	p-value^c^
**Individual**			
Age Group	1504		***<0.0001***
0–5 years	416	24.02 (12.92–44.65)	
6–10 years	250	6.26 (3.11–12.62)	
11–15 years	157	3.66 (1.60–8.39)	
>15 years	681	1.00 (baseline)	
Gender			
Female	854	1.00 (baseline)	***0.001***
Male	632	1.83 (1.28–2.61)	
Presence of *C. trachomatis* infection by PCR	258 (17.2)	11.19 (7.64–16.40)	***<0.0001***
Presence of ocular discharge	84 (5.6)	8.05 (4.62–14.02)	***<0.0001***
Presence of nasal discharge	261 (17.4)	7.90 (5.14–12.16)	***<0.0001***
Presence of flies on the face	21 (2.6)	2.188 (0.64–7.48)	*0.212*
**Household**			
Gender of household head (female)	509 (35.1)	0.99 (0.64–1.53)	*0.975*
Household size^d^		1.04 (0.99–1.09)	*0.068*
Number of children <10 years in household^d^		1.13 (1.02–1.24)	*0.019*
*Socio-economic status*			
Access to radio	747 (49.6)	0.94 (0.62–1.44)	*0.786*
Access to transport	227 (15.1)	1.20 (0.67–2.12)	*0.541*
Access to mobile phone	635 (42.2)	0.69 (0.45–1.06)	*0.090*
Relatives living in Bissau or abroad	1069 (71.0)	0.68 (0.43–1.07)	*0.090*
Access to savings at the end of the month	79 (5.3)	0.78 (0.28–2.13)	*0.623*
Educational level of household head			*0.298*
None	883 (58.7)	1.00 (baseline)	
Primary	466 (31.0)	1.31 (0.83–2.07)	
Secondary and above	156 (10.2)	0.76 (0.36–1.60)	
School attendance in children of school age	254 (62.4)	0.48 (0.30–0.76)	***0.002***
Health education received^e^	1054 (70.0)	0.75 (0.48–1.17)	*0.201*
*Water access*			
Access to water all year	1475 (97.5)	1.08 (0.23–4.99)	*0.923*
Access to water			***0.0002***
Multiple water sources	308 (20.5)	1.00 (baseline)	
Single water source other than natural spring	913 (60.7)	1.44 (0.87–2.54)	
Traditional natural spring only	284 (18.9)	3.37 (1.78–6.37)	
Water use within the household^e^^,^ f			*0.359*
<1 ‘vasilha’	67 (4.5)	2.29 (0.40–12.93)	
1 ‘vasilha’	145 (10.0)	3.59 (0.85–15.13)	
>1 ‘vasilha’	1283 (85.8)	2.91 (0.79–10.78)	
*Latrine access*			
Access to latrine	608 (40.4)	1.01(0.66–1.55)	*0.951*
Access to private latrine (vs shared)	284 (18.8)	0.98 (0.75–1.29)	*0.903*
Latrine use^e^			*0.450*
Always	500 (85.3)	0.90 (0.57–1.42)	
Sometimes	51 (8.7)	1.00 (0.32–3.20)	
Never	35 (6.0)	2.53 (0.75–8.54)	
Latrine cleanliness^g^			*0.471*
Very Clean – 1	33 (6.0)	0.24 (0.03–2.30)	
2	80 (14.6)	0.49 (0.16–1.50)	
3	274 (50.0)	1.09 (0.63–1.89)	
4	64 (11.7)	1.24 (0.46–3.32)	
Very Dirty - 5	97 (17.7)	1.48 (0.68–3.25)	
Flies (≥20) present around latrine^g^	251 (16.7)	1.26 (0.74–2.1)	*0.391*
Faeces visible in latrine^g^	200 (13.3)	0.89 (0.48–1.65)	*0.703*
*Environment* h			
Flies (≥20) present	510 (32.6)	1.08 (0.69–1.69)	*0.726*
Faecal waste (human/animal)	1105 (73.4)	1.07 (0.66–1.73)	*0.797*
Domestic waste	1175 (78.1)	0.97 (0.58–1.65)	*0.922*
Animals present	1298 (86.3)	0.66 (0.38–1.17)	*0.159*

a Active trachoma defined as TF (inflammatory trachoma-follicular) and/or TI (inflammatory trachoma-intense) using the WHO simplified scoring system [23].

b Unadjusted (crude) Odds Ratio (cOR) from two-level univariable mixed effects logistic regression analyses; CI = confidence interval.

c p-value for Wald test (Wald's Chi^2^); significant associations (where *p≤0.05*) are highlighted in bold.

d Continuous numeric variables.

e reported by household head.

f a ‘vasilha’ is a vessel of capacity ∼30 litres.

g researcher observed.

h researcher observed within 15 m of the household.

**Table 4:** Multilevel multivariable random effects logistic regression analysis of factors independently associated with active trachoma^a^.

		Model 1^b^		Model 2^c^ (Final Model)	
Variable	n	aOR(95%CI)^d^	p-value^e^	aOR (95% CI)^d^	p-value^e^
**Individual**					
Age Group	1504		***<0.0001***		***<0.0001***
0–5 years	416	13.66 (7.06–26.4)		10.22 (5.13–20.40)	
6–10 years	250	3.60 (1.70–7.59)		1.93 (0.88–4.24)	
11–15 years	157	3.08 (1.32–7.21)		2.41 (0.93–6.22)	
>15 years	681	1.00 (baseline)		1.00 (baseline)	
Gender					
Female	854	1.00 (baseline)	***0.034***	1.00 (baseline)	***0.005***
Male	632	1.58 (1.03–2.41)		1.89 (1.22–2.94)	
Presence of *C. trachomatis* infection	258	NA	NA	11.18 (6.9–18.1)	***<0.0001***
Presence of ocular discharge	84	2.71 (1.39–5.29)	***0.003***	2.04 (1.04–3.99)	***0.036***
Presence of nasal discharge	261	2.26 (1.35–3.77)	***0.002***	2.54 (1.51–4.26)	***0.001***
**Household**					
Access to water			***0.005***		***0.036***
Multiple water sources	308	1.00 (baseline)		1.00 (baseline)	
Single water source other than natural spring	913	1.42 (0.70–2.87)		0.90 (0.46–1.75)	
Traditional natural spring only	284	3.34 (1.50–7.45)		1.86 (0.89–3.89)	

a Active trachoma defined as TF (inflammatory trachoma-follicular) and/or TI (inflammatory trachoma-intense) using the WHO simplified scoring system [23].

b Model 1 shows the association of predictor variables with active trachoma.

c Model 2 includes the presence of *C. trachomatis* infection and demonstrates the effect of its inclusion on the predictor variables. Some of the association in Model 1 is mediated by *C. trachomatis* infection.

d Adjusted Odds Ratio (aOR) using multivariable two-level mixed effects logistic regression modelling; CI = confidence interval.

e p-value for Wald test (Wald's Chi^2^); significant associations (where *p≤0.05*) are highlighted in bold.

### Factors Associated with *C. trachomatis* Infection

Univariable associations with *C. trachomatis* infection are presented in *Table 5*. In the final multivariable model *C. trachomatis* infection was strongly independently associated with being aged ≤10 years. The presence of ocular discharge (OR = 2.3 (95% CI 1.3–4.4)) and household access only to a traditional natural spring (OR = 6.6 (95% CI 2.8–15.2)) and or access to a single water source only (OR = 3.9 (95% CI 1.9–8.0)) (rather than households who had access to multiple water sources) were strongly associated with infection (*Table 6*). The presence of flies around a latrine was also independently associated with infection (OR = 2.1 (95% CI 1.1–3.8)). The presence of flies around a latrine were strongly associated with the presence of flies in the environment surrounding the household (OR = 8.3 (95% CI 5.4–12.7), *p<0.0001*) and the presence of visible faeces within the latrine (OR = 46.7 (95% CI 28.5–76.6), *p<0.0001*). There was no association between flies in the environment (OR = 1.1 (95% CI 0.4–3.0), *p = 0.91*) nor flies around the latrine (OR = 0.5 (95% CI 0.1–2.6), *p = 0.43*) with flies on the face at the time of examination.

**Table 5:** Multilevel univariable random effects logistic regression analysis of factors associated with ocular *C. trachomatis* infection.

Variable	n	cOR (95% CI)^a^	*p-value* b
**Individual**			
Ethnicity	1497		***0.004***
Bijagos	1395	5.05 (1.68–15.17)	
Other	102	1.00 (baseline)	
Age Group	1504		***<0.0001***
0–5 years	416	3.28 (2.24–4.80)	
6–10 years	250	4.03 (2.59–6.29)	
11–15 years	157	1.58 (0.90–2.78)	
>15 years	681	1.00 (baseline)	
Gender			
Female	854	1.00 (baseline)	*0.753*
Male	632	1.05 (0.77–1.44)	
Presence of ocular discharge	84 (5.6)	4.17 (2.35–7.38)	***<0.0001***
Presence of nasal discharge	261 (17.4)	2.17 (1.48–3.20)	***<0.0001***
Presence of flies on the face	21 (2.6)	2.00 (0.57–7.10)	*0.282*
**Household**			
Gender of household head (female)	509 (35.1)	0.79 (0.48–1.29)	*0.343*
Household size^c^		1.04 (0.99–1.10)	*0.127*
Number of children <10 years in household^c^		1.09 (0.97–1.22)	*0.132*
*Socio-economic status*			
Access to radio	747 (49.6)	0.98 (0.61–1.58)	*0.945*
Access to transport	227 (15.1)	1.14 (0.58–2.24)	*0.699*
Access to mobile phone	635 (42.2)	0.57 (0.35–0.92)	*0.022*
Relatives living in Bissau or abroad	1069 (71.0)	0.88 (0.52–1.58)	*0.643*
Access to savings at the end of the month	79 (5.3)	0.69 (0.22–2.16)	*0.527*
Educational level of household head			*0.200*
None	883 (58.7)	1.00 (baseline)	
Primary	466 (31.0)	1.26 (0.75–2.12)	
Secondary and above	156 (10.2)	0.56 (0.24–1.30)	
School attendance in children of school age	254 (62.4)	0.82 (0.56–1.19)	*0.289*
Health education received^d^	1054 (70.0)	0.78 (0.47–1.30)	*0.346*
*Water access*			
Access to water all year	1475 (97.5)	0.75 (0.15–3.91)	*0.734*
Access to water			***<0.0001***
Multiple water sources	308 (20.5)	1.00 (baseline)	
Single water source other than natural spring	913 (60.7)	3.69 (1.85–7.38)	
Access to natural spring only	284 (18.9)	7.01 (3.11–15.81)	
Water use within the household^d^^,^ e			*0.776*
<1 ‘vasilha’	67 (4.5)	2.21 (0.47–10.43)	
1 ‘vasilha’	145 (10.0)	1.41 (0.39–5.02)	
>1 ‘vasilha’	1283 (85.8)	1.56 (0.54–4.53)	
*Latrine access*			
Access to latrine	608 (40.4)	1.21 (0.75–1.97)	*0.435*
Access to latrine (private vs shared)	284 (18.8)	1.03 (0.76–1.39)	*0.840*
Latrine use^d^			*0.321*
Always	500 (85.3)	1.10 (0.67–1.80)	
Sometimes	51 (8.7)	0.66 (0.17–2.58)	
Never	35 (6.0)	3.73 (0.84–16.6)	
Latrine cleanliness^f^			*0.228*
Very Clean – 1	33 (6.0)	0.55 (0.09–3.32)	
2	80 (14.6)	0.58 (0.19–1.76)	
3	274 (50.0)	1.08 (0.59–1.98)	
4	64 (11.7)	1.11 (0.35–3.48)	
Very Dirty - 5	97 (17.7)	2.63 (1.13–6.12)	
Flies (≥20) present around latrine^f^	251 (16.7)	1.90 (1.05–3.41)	***0.033***
Faeces visible in latrine^f^	200 (13.3)	1.15 (0.59–2.24)	*0.687*
*Environment* g			
Flies (≥20) present	510 (32.6)	1.37 (0.84–2.22)	*0.209*
Faecal waste (human/animal)	1105 (73.4)	0.90 (0.53–1.54)	*0.705*
Domestic waste	1175 (78.1)	0.65 (0.36–1.17)	*0.150*
Animals present	1298 (86.3)	0.49 (0.26–0.94)	***0.030***

a Unadjusted (crude) Odds Ratio (cOR) using two-level univariable mixed effects logistic regression; CI = confidence interval.

b p-value for Wald test (Wald's Chi^2^); significant associations (where *p≤0.05*) are highlighted in bold.

c Continuous numeric variables.

d reported by household head.

e a ‘vasilha’ is a vessel of capacity ∼30 litres.

f researcher observed.

g researcher observed within 15 m of the household.

**Table 6:** Multilevel multivariable random effects logistic regression analysis of factors associated with ocular *C. trachomatis* infection.

Variable	n	aOR (95% CI)^a^	p-value^b^
**Individual**			
Age Group	1504		***<0.0001***
0–5 years	416	3.10 (1.04–4.70)	
6–10 years	250	3.83 (2.38–6.16)	
11–15 years	157	1.65 (0.92–2.96)	
>15 years	681	1.00 (baseline)	
Gender			*0.377*
Female	854	1.00 (baseline)	
Male	632	0.86 (0.61–1.21)	
Presence of ocular discharge	84	2.33 (1.25–4.35)	***0.007***
**Household**			
Access to water			***<0.0001***
Multiple water sources	308	1.00 (baseline)	
Single water source other than natural spring	913	3.88 (1.88–8.01)	
Traditional natural spring only	284	6.57 (2.83–15.23)	
Flies (≥20) present around latrine^c^	251	2.06 (1.10–3.84)	***0.023***

a Adjusted Odds Ratio (aOR) using two-level multivariable mixed effects logistic regression modelling; CI = confidence interval.

b p-value for Wald test (Wald's Chi^2^); significant associations (where *p≤0.05*) are highlighted in bold.

c researcher observed.

## Discussion

We have shown that there is clustering of both active trachoma and infection at household, village and island levels. We found that the most substantial clustering was present at household-level, thus the random effects introduced by this are adjusted for using two-level hierarchical mixed effects models. Clustering at household level has been described in other populations [10], [13], [29]. We found *C. trachomatis* infection to be more strongly clustered at the household level than active trachoma, which has also been reported in Tanzanian communities [18].

Consistent with results from other studies, we found that being aged 0–5 years [10], [16], [29], [30]–[34], the presence of ocular *C. trachomatis* infection [17] and the presence of ocular and nasal discharge [19], [32], [35], [36] were strongly associated with active trachoma. We also found that being female was associated with a reduced odds of active trachoma, but not infection. The finding of an association between gender and active trachoma are contradictory in the literature [36]–[38]. In this analysis gender was included in all models due to *a priori* assumptions.

At the household level, the only household-level factor independently associated with both active trachoma and *C. trachomatis* infection was access to type and number of water sources, where household access to a traditional natural spring as the only water source was more strongly associated with active trachoma and *C. trachomatis* infection than access to another (single) source or multiple water sources of various types. This association is likely to be mediated through the presence of *C. trachomatis* infection. A similar association was shown in Ethiopia, where a piped water supply was associated with reduced risk of active trachoma compared to spring or river/lake water sources [37]. This may have been related to increased distance to the non-piped water sources [34] or to volume of water used [33]. In Bijagós communities, every household has access to a water source within 15 minutes walking distance. It is likely that this association may reflect more subtle hygiene and water use behaviours related to allocation of water collected for specific hygiene practices. A greater volume of water can be obtained with greater ease from an improved well or borehole than is possible from a natural spring. Although we found no statistically significant association between the volume of water stored in the household and active trachoma, it may be that the volume of water specifically allocated for hygiene practices is important, as shown in The Gambia [39], and that this finding is a proxy for water use allocation.

Children aged 0–5 years are at increased risk of ocular infection with *C. trachomatis*. In 6–10 year olds the risk is similarly elevated. In contrast, children aged 0–5 years are at much greater risk of active trachoma than 6–10 year olds. This supports suggestions that young children are the main reservoir of infection [10], [19], [32] and demonstrates that this reservoir persists beyond pre-school age children in this population. The difference found in associations between age and active trachoma and infection may be explained by the presumed acquisition of immunity in childhood, which may result in reduced prevalence of clinically active trachoma despite the remaining strong association with infection. Duration [40] and intensity [41] of active trachoma and *C. trachomatis* infection have been shown to be age-dependent. It may also be that clinical signs present in 0–5 year olds are less specific for *C. trachomatis* infection and that other bacteria such as *Streptococcus pneumoniae* and *Haemophilus influenzae* contribute to the clinical phenotype, such that they induce a recall of the follicular response in children previously exposed to *C. trachomatis*, as hypothesised in cross-sectional studies showing an association between follicular trachoma and these bacteria in Tanzania and The Gambia [42], [43]. Overall, there is a diminishing association with age, indicating that age may also represent a proxy for close contact and specific hygiene behaviours, acquired immunity and duration of infection. Whilst there were strong univariable associations between infection and ocular and nasal discharge, the association with nasal discharge is not significant in the multivariable model. Ocular discharge may more specific for *C. trachomatis* infection than nasal discharge, likely representing a consequence of *C. trachomatis* infection rather than a risk factor for it.

Latrine access was not associated with active trachoma or *C. trachomatis* infection, despite the fact that 60% of individuals do not have access to a latrine. Latrine access does not translate into latrine use [44]. We assessed latrine use through observation of the latrines in addition to collecting reported data. Although there is high reported use of latrines within a household with latrine access, there may be household members that do not use the latrine. In these populations children aged 1–9 years are often discouraged from using latrines due to caregiver concerns about their safety when using latrines (Thompson *et al.*, unpublished data).

The presence of flies around a household pit latrine was strongly associated with infection in the final multivariable model, although there was no association between flies on the faces of individuals at the time of examination with either active trachoma or infection. The presence of flies on faces has been well-documented as a risk factor for active trachoma in some environments [8], [29], [45] and there is evidence that the eye-seeking flies *Musca sorbens* (and possibly other domestic muscidae) are passive vectors in *C. trachomatis* transmission [45]–[48]. One study that examined fly density around used household pit latrines in The Gambia found that the majority of flies emerging from the latrine were *Chrysomya albiceps*, rather than *M. sorbens* [47]. We do not have data on the species of flies around pit latrines in Bijagós communities, however it is possible that there is ovipositioning by *M. sorbens* in or around latrines in these communities. We found a strong association between flies present in the environment and flies around the latrine, although there was no association with flies on the face at the time of examination. With decreasing age, there was a trend in association between flies on the face and flies around the latrine, but this association was non-significant.

*M. sorbens* have been shown to preferentially breed in human excreta, in addition to domestic animal faeces [46]. Although the presence of animal and human excreta within 15 metres of the household were not risk factors for active trachoma or *C. trachomatis* infection, they were ubiquitous in the villages. There was a strong association noted between flies around a latrine and the presence of visible faeces within a latrine. These findings suggest that flies may be an important in the transmission of trachoma in this environment. Our study was conducted in the cooler dry season, possibly explaining the lack of association between flies on faces and in the environment and *C. trachomatis* infection in the regression analysis. Fly populations and density have been shown to have seasonality elsewhere [49], [50].

The cross-sectional design of this study is a limitation in understanding determinants of disease and infection. There may also be some limitations in using stepwise regression methods such that models may be overfitted or unstable [51]. Risk factors are often difficult to define and despite the presence of statistically significant associations, it is likely that the relationship between socio-behavioural and environmental risk factors and disease and infection is complex and not fully explained by this analysis. It may be that intra- and inter-familial transmission occurs in these communities as a consequence of specific hygiene practices and complex socio-behavioural factors. These factors are often difficult to define and require longitudinal studies beyond the scope of the current study. Further epidemiological studies examining the species and contribution of flies to trachoma transmission and water use and specific hygiene behaviours in relation to trachoma and *C. trachomatis* infection in this population may be of importance in successful trachoma control activities.

### Conclusion

We have described individual and household-level risk factor associations with active trachoma and ocular infection with *C. trachomatis* on the Bijagós Archipelago to improve our understanding of the relationship between disease and infection in this remote treatment-naïve trachoma-hyperendemic population.

These data suggest that in this environment household-level risk factors relating to fly populations, hygiene behaviours and water usage are likely to be important in the transmission of ocular *C. trachomatis* infection. Education about cleanliness, sanitation and hygiene practices is likely to be important in reducing transmission of infection in these communities. Ensuring the provision of water sources which allow adequate water to be allocated for hygiene may assist this, and further studies examining specific hygiene practices may be useful. Reducing fly populations around the latrines where they exist may be of benefit. These findings may be important in the implementation of the F and E components of SAFE in this population.

In order to fully understand the factors associated with active trachoma and ocular *C. trachomatis* infection in these communities, further epidemiological studies examining transmission and clustering of *C. trachomatis* infection are required. These studies should focus on pathogen factors such as the role of infection intensity and strain diversity, and socio-behavioural factors such as specific hygiene behaviours.

## Supporting Information

Checklist S1 STROBE checklist.
(DOCX)
